# Probing the reversibility and kinetics of Li^+^ during SEI formation and (de)intercalation on edge plane graphite using ion-sensitive scanning electrochemical microscopy†
†Electronic supplementary information (ESI) available: Experimental methods, further simulation information, and additional experimental data. See DOI: 10.1039/c9sc03569a


**DOI:** 10.1039/c9sc03569a

**Published:** 2019-10-08

**Authors:** Zachary T. Gossage, Jingshu Hui, Yunxiong Zeng, Heriberto Flores-Zuleta, Joaquín Rodríguez-López

**Affiliations:** a Department of Chemistry , University of Illinois at Urbana-Champaign , 600 S Mathews Ave. , Urbana , Illinois 61801 , USA . Email: joaquinr@illinois.edu ; Tel: +1-217-300-7354

## Abstract

Ions at battery interfaces participate in both the solid-electrolyte interphase (SEI) formation and the subsequent energy storage mechanism. However, few *in situ* methods can directly track interfacial Li^+^ dynamics. Herein, we report on scanning electrochemical microscopy with Li^+^ sensitive probes for its *in situ*, localized tracking during SEI formation and intercalation. We followed the potential-dependent reactivity of edge plane graphite influenced by the interfacial consumption of Li^+^ by competing processes. Cycling in the SEI formation region revealed reversible ionic processes ascribed to surface redox, as well as irreversible SEI formation. Cycling at more negative potentials activated reversible (de)intercalation. Modeling the ion-sensitive probe response yielded Li^+^ intercalation rate constants between 10^–4^ to 10^–5^ cm s^–1^. Our studies allow decoupling of charge-transfer steps at complex battery interfaces and create opportunities for interrogating reactivity at individual sites.

## Abstract 2

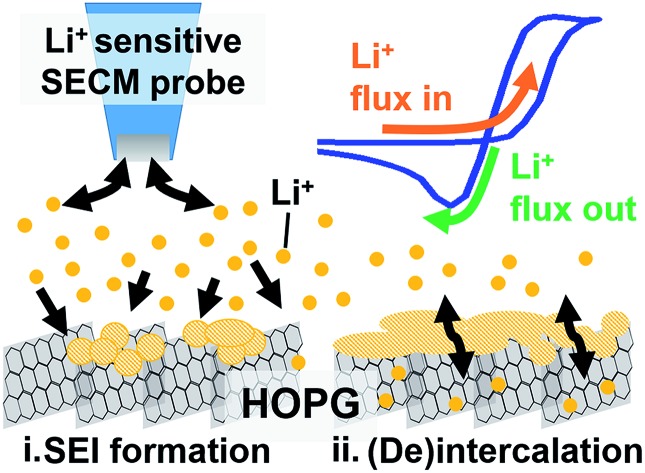
Tracking down Li^+^ flux during complex ion intercalation processes on a battery interface.

## Introduction

Understanding fundamental charge transfer at interphases is a research priority for enabling better energy storage technologies.^1^–^3^ In high energy density anodes, such as carbon and silicon, heterogeneous charge-mediating interphases determine electrode cycling performance, materials utilization, and risk for failure.^1^ The solid electrolyte interphase (SEI) plays a major role in the ability of lithium-ion batteries (LIBs) to operate in a reliable manner.^1^,^2^ The SEI components and properties are derived from electrolyte decomposition reactions at the surface of the anode, resulting in a morphologically and chemically heterogeneous structure.^2^,^4^–^7^ Materials characterization methods have led to improved understanding of the components and precursors involved in the SEI,^1^,^2^,^8^,^9^ on the observation of its reactivity and morphological changes during formation,^10^,^11^ and tracking of the intercalation process.^12^–^14^ On the other hand, there are few *in situ* methods capable of tracking interfacial alkali ions (*e.g.* Li^+^)^15^ and the impact of SEI progressive growth on their response.

Ions at the electrode–electrolyte interface play a key role in both SEI formation and the subsequent energy storage mechanism. Thus, structural heterogeneity may lead to reactive heterogeneity, ultimately affecting local ionic fluxes and cycling performance at differentiated sites.^16^,^17^ Several groups have successfully relied on tracking atomic states or phase change to infer Li^+^ movement throughout bulk electrode materials,^13^,^14^,^18^,^19^ but the extension of this analysis to the SEI is not easily attainable due to its thickness (typically <100 nm), variable molecular content, and amorphous nature.^1^ Ultimately, direct and localized quantification of Li^+^ is desirable to provide key insight into ion intercalation kinetics, the ion diffusion mechanism through the SEI, localized heterogeneities, and SEI dynamics during charge/discharge.

The unique aspect of the analytical approach presented here comes from accurately measuring the local Li^+^ response^20^ as SEI formation and (de)intercalation reactions occur at the anode.^21^–^23^ Emerging ion-sensitive scanning probe methods (SPMs) show great potential for understanding processes at functioning electrodes to guide development of next-generation energy storage technologies.^2^,^24^–^26^ Scanning electrochemical microscopy (SECM) is a highly versatile SPM that is capable of acquiring both ionic and electronic information at an electrode surface within real battery environments.^2^,^27^ However, quantitative ionic measurements require specialized probes and are far less common among SECM studies.^21^,^23^,^24^ Recently, our group applied Hg probes to detect ion fluxes into multi-layered graphene (MLG)^23^ and patterned highly-oriented pyrolytic graphite (HOPG).^21^ Recent work in our lab regarding probe fabrication and positioning^22^ has dramatically improved their performance, enabling exciting directions in the exploration of ion dynamics on activated battery electrodes.

**Fig. 1:**
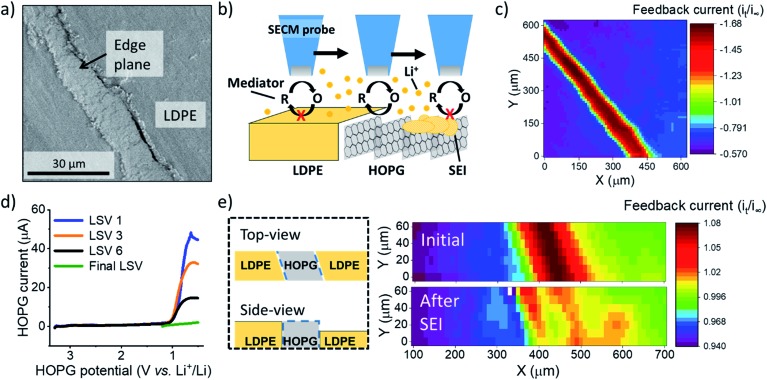
SEI formation on the HOPG edge plane. (a) Scanning electron microscopy of an unused region of HOPG edge. (b) Illustration of experimental setup and procedure for SECM imaging and positioning. (c) SECM feedback image of the HOPG substrate. (d) Multiple LSV sweeps on HOPG in the SEI region. (e) SECM images before and after SEI formation. A diagram of the sample (on left) represent the HOPG sample geometry; explaining the tilt observed in the SECM images. SECM and LSV were collected in 0.1 M LiClO_4_, PC : EC (1 : 1) with 15 mM Fc. For the SECM images, the measured current, *i*_t_, was normalized by the limiting current far from the substrate, *i*_∞_.

In this work, we used redox and ion-sensitive modes of SECM to track Li^+^ flux during SEI formation at the edge site of HOPG. HOPG is a model carbon material that enables the straightforward selection of the Li^+^ intercalation sites, *i.e.* the edge plane, for its characterization.^28^–^30^ We used HOPG substrates (Fig. 1a) with the edge plane sealed between two pieces of low-density polyethylene (LDPE) as described in the ESI (Section 1 and 3; Fig. S1 and S2†). The edge plane is the predominant site for (de)intercalation in graphitic materials,^31^–^33^ showing high electron transfer kinetics^34^ and high Li^+^ site density^29^ compared with the basal plane. Also, the edge plane contains functional groups and defects capable of interacting with Li^+^.^35^–^37^ Few reports studied the edge plane using electrochemistry coupled to structural imaging using SPMs such as atomic force and scanning tunneling microscopy, and spectroscopy.^29^,^30^,^38^–^40^ These studies provided substantial insight into the intercalation process of predominant edge and basal plane electrodes and the effect of various electrolytes and additives. However, there remains limited information regarding interfacial processes from the viewpoint of ionic species, in contrast to changes in the host material. Direct inspection of ion-related phenomena, such as intercalation kinetics, and ion-coupled redox processes, is key to understanding the complexity of the battery interphase.

## Results and discussion

We immersed an HOPG edge plane in a mixed propylene carbonate and ethylene carbonate (PC : EC (1 : 1 ratio by volume)) electrolyte containing 100 mM lithium perchlorate (LiClO_4_) and 15 mM ferrocene (Fc) as a redox mediator to probe the local electron transfer kinetics with imaging (Fig. 1b and c). Once the probe was approached to the surface, we observed characteristic mass transfer limited positive feedback (increased redox response) on the SECM probe when transiting above the conductive HOPG (Fig. 1c). In contrast, the insulating LDPE showed a characteristic negative feedback (decreased current, Fig. 1c, S3 and S4†). This provided clear identification of the edge location for further positioning in other experiments. We used linear sweep voltammetry (LSV) to form the SEI (Fig. 1d). Previous reports indicated that SEI formation on carbon occurs on a wide potential window preceding bulk intercalation, which begins at potentials <0.3 V.^23^,^41^–^43^ Hereon, we identify these two electrode potential regions as the SEI and intercalation regions.^23^

We first focus on the SEI region. In the first sweep, a cathodic wave peaked near 1.1 V in the HOPG response (Fig. 1d). Upon further sweeps, this cathodic wave diminished suggesting a passivation process.^23^,^29^ SECM imaging also indicated significant passivation, as evidenced by a decreasing feedback current; however significant heterogeneity was also observed, suggesting differences in the local electron transfer kinetics (Fig. 1e). We observed similar features and an increase in roughness with SEM after SEI and intercalation experiments (Fig. S5†). All results suggested SEI formation at the HOPG edge, alike to that observed on other graphitic samples.^7^,^23^,^44^

**Fig. 2:**
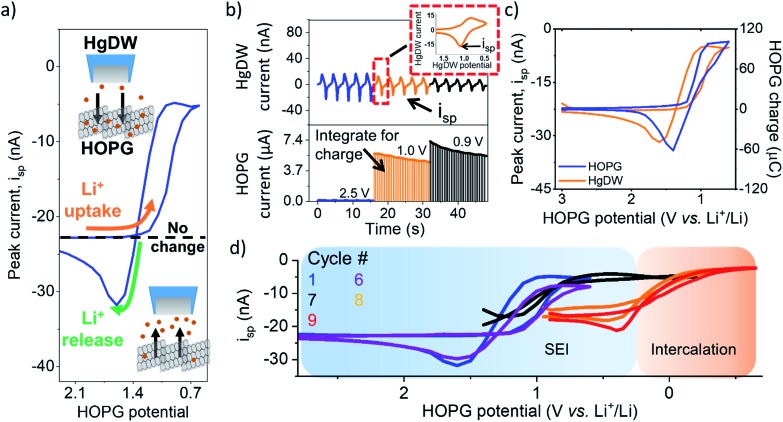
Li^+^ flux at HOPG during SEI formation. (a) Illustration of processed measurements. (b) Process for collecting the data by cycling the HgDW at 1 V s^–1^ (top) while controlling the HOPG potential (bottom). The inset shows a single HgDW cycle with current *vs.* HgDW potential. The charge for each transient at HOPG was determined through integration at each potential. (c) Comparison of extracted peak currents, *i*_sp_, from the HgDW and the integrated HOPG response for each potential during the 1^st^ SEI formation cycle. (d) Measured *i*_sp_ during cycling in the SEI and Intercalation regions. Cycles 1 and 6 are normalized based on the linear region >1.5 V.

To analyze changes in Li^+^ flux during the SEI formation process, we focused on an electrolyte containing 10 mM lithium hexafluorophosphate (LiPF_6_) as the Li^+^ source and 100 mM tetrabutyl ammonium hexafluorophosphate (TBAPF_6_) supporting electrolyte. Batteries commonly involve at least 1 M Li^+^ concentrations to maintain high conductivity and accommodate loss of Li^+^ during SEI formation and cycling. However, these conditions are not strict limitations for SEI formation and Li^+^ intercalation.^23^,^45^ Following detection of the edge-plane using SECM feedback (Fig. S6†), we rinsed the cell from the redox mediator and switched to a mercury disc-well (HgDW, Fig. S7†) for measuring the Li^+^ response.^22^ This was accomplished by continuous cycling of the probe at 1 V s^–1^ under conditions of stripping voltammetry, thus detecting local depletion and enrichment^46^,^47^ of ions upon activation of the HOPG substrate, as depicted in Fig. 2a and b. We monitored changes in the stripping peak current (*i*_sp_, Fig. 2a and b) as a direct indicator of the local Li^+^ concentration resulting from the ion flux to the electrode.^20^,^21^,^23^ Inward and outward fluxes were detected by *i*_sp_, with Li^+^ consumption by the HOPG electrode decreasing the absolute value of *i*_sp_, and *vice versa*.

Focusing on the SEI formation region (Fig. 2c),^23^ we decreased the potential of the HOPG electrode in 100 mV increments from 3.0 V to 0.6 V *vs.* Li^+^/Li (Fig. S8†) in a similar fashion to the potentiometric intermittent titration technique, or PITT.^48^,^49^ To better compare the probe and substrate responses over the step interval, we integrated the current passed by the substrate during each increment (Fig. 2b, bottom) to yield an HOPG charge (Fig. 2c). During the forward sweep, and especially when stepping more negative than 1.3 V, we observed a concurrent cathodic process on the HOPG and a decrease in *i*_sp_ (Fig. 2c). This potential range agrees with previous reports for irreversible SEI formation on graphitic and edge plane electrodes.^10^,^29^,^50^ The decrease in *i*_sp_ follows the trend of the HOPG response indicating that Li^+^ is being consumed by HOPG as part of the electrochemical reaction during the cathodic sweep. Interestingly, stepping the HOPG again positive reversed this trend, revealing an anodic process at 1.4 V and the concurrent increase in *i*_sp_ implying a reversible process involving an outward flux of Li^+^.

The edge plane has a high density of Li^+^ sites^29^ and can be a site of disorder^51^ and functional groups.^37^ Previous reports on HOPG suggested Li^+^ insertion as part of the SEI formation mechanism;^29^,^43^,^50^ on the other hand, Li^+^ intercalation occurs at more negative potentials, below 0.3 V.^29^,^52^ A reversible SEI film was reported on HOPG as long as potentials were kept positive of 1 V *vs.* Li^+^/Li.^6^ Further, redox-active organic groups involving carbonyl species at the edge plane can cause a flux of Li^+^; in this case, Li^+^ uptake into the SEI would result from reduction of charge neutral C–O groups to the negatively charged species, thus binding to the positively charged alkali.^37^,^53^,^54^ Following the method proposed in the works of McCreery,^55^,^56^ we used 2,4-dinitrophenylhydrazine (DNPH) as a Raman-active molecular tag for carbonyl functionality at the electrode surface. This experiment indeed showed the presence of surface carbonyl groups on the original samples; by comparing the peak areas for graphite and DNPH Raman peaks and assuming a flat surface, we estimate >90% coverage by carbonyl groups at the HOPG edge (Fig. S9†). Evaluation of the anodic charge passed upon voltammetric scan reversal (Fig. S10†) indicated a charge density of 110 mC cm^–2^ for the reversible species, suggesting the formation of a multi-layer of redox-active material. We speculate that the observed flux at this potential region results from reversible Li^+^ insertion and deinsertion during redox of this SEI, as Li^+^ release was dependent on the history of the sweep, *i.e.* only observed after a reduction process had taken place and at sufficiently positive HOPG potentials (>1.4 V *vs.* Li^+^/Li). Another possibility is that the formation of a dynamic SEI, *e.g.* dissolving after formation, could lead to the observed Li^+^ release.^6^ Regardless of the origin, these results suggest a direct observation of the reversible nature of an SEI on the graphitic edge plane.

We noted a significant capacity loss (60%) during the first cycle between the forward/reverse sweeps (Fig. 2c and S11†) suggesting simultaneous reversible and irreversible components to the total process. Both the cathodic and anodic processes occurring at HOPG decreased upon further cycling (Fig. S11†) evidencing the transient formation of the SEI. These changes in behavior were mirrored on the probe response (Fig. 2d), which showed a smaller change in *i*_sp_ with cycle number at a fixed potential window (*e.g.* cycle 1–6). Polarizing further negative had the effect of consuming these SEI formation processes and gave way to new ones.

Next, we focus on the intercalation region (Fig. 2d). After a brief transient where the HOPG and HgDW responses revealed further SEI formation (Fig. 2d, cycle 7), we observed the onset of a second cathodic process (<0.3 V). Stepping the HOPG potential further negative (Fig. 2d, cycles 8 and 9) revealed a cathodic plateau (<0.3 V) paired with an anodic peak (0.4 V) on the return. This behavior is consistent with Li^+^ uptake during intercalation, and a release, enriching Li^+^ local concentration, during deintercalation. We note also that the potential region for this second process agrees with (de)intercalation at bulk graphite.^41^,^57^ Also, the reversible SEI process (>0.6 V) quickly diminished upon sweeping further negative, leaving behind only a slightly decreased signal on the idle *i*_sp_ background, *e.g.* compare the origin of cycles 1 and 9 on Fig. 2d. This transition has rarely been discussed,^6^ though HOPG and its edge plane undergo substantial structural changes during SEI formation and cycling.^58^ We also observed a similar transition with a high Li^+^ concentration (Fig. S10 and S12†). Despite the fundamental differences between processes such as intercalation and SEI formation, our SECM approach is capable of detecting the resulting ion responses as the substrate is activated.

**Fig. 3:**
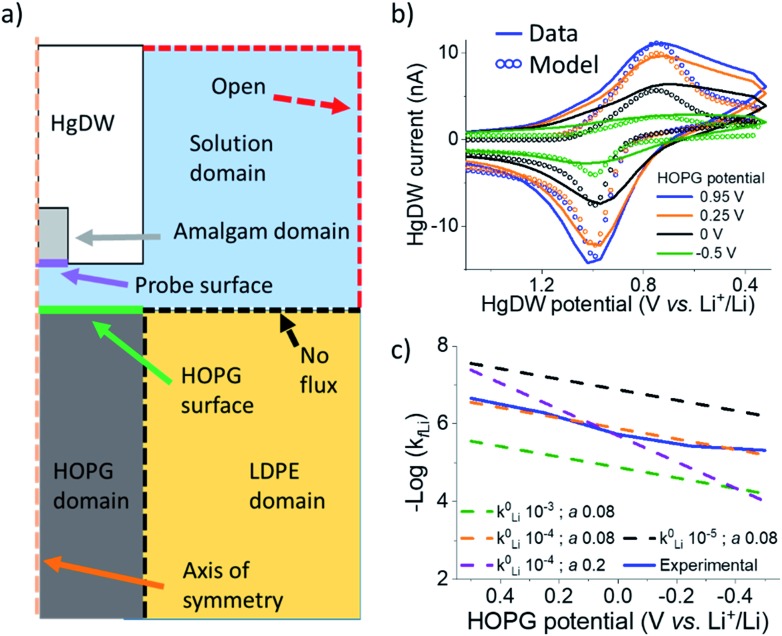
COMSOL modeling of intercalation kinetics. (a) Diagram of the COMSOL model for determining *k*_*f*Li_. (b) Fitting of probe response with a COMSOL model for the intercalation in cycle 8. (c) Total data set for the cycle 8 and extracted *k*_*f*Li_ fit to different *k*0Li and α based on B–V.

We now turn to quantifying the local intercalation kinetics aided by the measurement of *i*_sp_. By using COMSOL Multiphysics finite element method, we simulated the probe response in a 2D axisymmetric geometry (Fig. 3a) during the intercalation sweep, assuming reported intercalation parameters on other graphitic materials as initial conditions (ESI Section 2†). We modified our previous model^22^ with an HOPG domain (Fig. 3a) that consumed Li^+^ based on a defined forward rate constant, *k*_*f*Li_, which caused a response on the simulated SECM tip voltammetry (Fig. 3b). *k*_*f*Li_ can be further understood in the context of Butler–Volmer kinetics (B–V), as is done in Fig. 3c, but it does not assume this model in the calculation of the values presented in Table 1. Therefore, these values can be used to understand fundamental activation aspects in more complex derivations of the graphitic system.^59^

**Table 1:** Extracted *k*_*f*Li_ from COMSOL fittings

HOPG potential (V *vs.* Li^+^/Li)	*k*_*f*Li_ (cm s^–1^)
0.5	2.8 × 10^–5^
0.25	6.1 × 10^–5^
0	1.3 × 10^–4^
–0.25	2.9 × 10^–4^
–0.5	6.3 × 10^–4^

By fitting the overall response, we determined a *k*0Li of 10^–4^ cm s^–1^ for the HOPG substrate. Our results in Fig. 3e are significantly faster than reported rates for (de)intercalation at bulk graphite (10^–7^ cm s^–1^),^41^ and agree more with electron transfer kinetics.^31^,^60^ Both electron transfer kinetics and the fraction of edge-to-basal plane are known to affect intercalation kinetics.^31^ Due to the low potentials accessed, plating may also have occurred aside intercalation. However, the HOPG response does not indicate plating or the familiar “cross-over” due to nucleation^61^ until stepping 150 mV further negative during cycle 9 (Fig. S14†). Aside, our best fit was for low α (Fig. 3c), suggesting our SEI or the edge itself have a pinhole-like structure and small kinetic domains.^60^,^62^ Accurate modeling of deintercalation kinetics would require detailed knowledge of bulk transport and state of charge at the HOPG electrode, however the change in *i*_sp_ observed for this process suggests faster rates (Fig. S13†) than for intercalation, consistent with previous reports.^63^–^65^ Kinetics related to the SEI formation process and intercalation are key parameters for battery performance and limitations.^59^,^66^ Our methods move away from bulk characterization of kinetics to measurements at a single location addressed by a versatile probe.

## Conclusions

In conclusion, our SECM approach was capable of correlating Li^+^ flux as the HOPG interface was activated toward both irreversible SEI formation and (de)intercalation. The highly reactive HOPG edge plane shows potential regime-dependent behavior. Cycling in a high potential region (>0.6 V) led to SEI formation, but also a rarely reported redox reaction involving reversible exchange of Li^+^.^37^ Upon stepping the HOPG further negative, we observed a transition to (de)intercalation. The HgDW response agreed with bulk measurements and captured local screenshots of Li^+^ uptake and release by the substrate. By developing a COMSOL model of the intercalation process, we determined localized, fundamental kinetic information. Our strategy paves the way toward *in situ*, kinetic mapping of ionic processes,^22^ smaller probes and higher resolution,^67^ and amenable chemical resolution for emerging next-generation ion batteries.^68^–^72^

## Conflicts of interest

There are no conflicts to declare.

## Supplementary Material

Supplementary information

## References

[cit1] Tripathi A. M., Su W.-N., Hwang B. J. (2018). Chem. Soc. Rev..

[cit2] Hui J., Gossage Z. T., Sarbapalli D., Hernandez-Burgos K., Rodríguez-López J. (2018). Anal. Chem..

[cit3] Crabtree G., Rubloff G. and Takeuchi E., Basic research needs for next generation electrical energy storage, Report of the Office of Basic Energy Sciences Workshop on Energy Storage, U.S. Department of Energy Office of Science, Washington, D.C., 2017.

[cit4] Song H.-Y., Jeong S.-K. (2018). J. Power Sources.

[cit5] Lu D., Tao J., Yan P., Henderson W. A., Li Q., Shao Y., Helm M. L., Borodin O., Graff G. L., Polzin B. (2017). Nano Lett..

[cit6] Seidl L., Martens S., Ma J., Stimming U., Schneider O. (2016). Nanoscale.

[cit7] Bülter H., Schwager P., Fenske D., Wittstock G. (2016). Electrochim. Acta.

[cit8] Olson J. Z., Johansson P. K., Castner D. G., Schlenker C. W. (2018). Chem. Mater..

[cit9] Shi F., Ross P. N., Somorjai G. A., Komvopoulos K. (2017). J. Phys. Chem. C.

[cit10] Cresce A. v., Russell S. M., Baker D. R., Gaskell K. J., Xu K. (2014). Nano Lett..

[cit11] Steinhauer M., Stich M., Kurniawan M., Seidlhofer B.-K., Trapp M., Bund A., Wagner N., Friedrich K. A. (2017). ACS Appl. Mater. Interfaces.

[cit12] Lopez J. L. L., Grandinetti P. J., Co A. C. (2018). J. Mater. Chem. A.

[cit13] Nowack L., Grolimund D., Samson V., Marone F., Wood V. (2016). Sci. Rep..

[cit14] Zhang K., Ren F., Wang X., Hu E., Xu Y., Yang X.-Q., Li H., Chen L., Pianetta P., Mehta A. (2017). Nano Lett..

[cit15] Yamanaka T., Nakagawa H., Tsubouchi S., Domi Y., Doi T., Abe T., Ogumi Z. (2017). ChemSusChem.

[cit16] Snowden M. E., Dayeh M., Payne N. A., Gervais S., Mauzeroll J., Schougaard S. B. (2016). J. Power Sources.

[cit17] Danis L., Gateman S. M., Kuss C., Schougaard S. B., Mauzeroll J. (2017). ChemElectroChem.

[cit18] Yu S.-H., Huang X., Schwarz K., Huang R., Arias T. A., Brock J. D., Abruña H. D. (2018). Energy Environ. Sci..

[cit19] Wang J., Chen-Wiegart Y.-c. K., Eng C., Shen Q., Wang J. (2016). Nat. Commun..

[cit20] Barton Z. J., Rodríguez-López J. (2014). Anal. Chem..

[cit21] Barton Z. J., Hui J., Schorr N. B., Rodríguez-López J. (2017). Electrochim. Acta.

[cit22] Barton Z. J., Rodríguez-López J. (2017). Anal. Chem..

[cit23] Hui J., Burgess M., Zhang J., Rodríguez-López J. (2016). ACS Nano.

[cit24] Barton Z. J., Rodríguez-López J. (2016). Anal. Bioanal. Chem..

[cit25] Balke N., Jesse S., Kim Y., Adamczyk L., Tselev A., Ivanov I. N., Dudney N. J., Kalinin S. V. (2010). Nano Lett..

[cit26] Takahashi Y., Kumatani A., Munakata H., Inomata H., Ito K., Ino K., Shiku H., Unwin P. R., Korchev Y. E., Kanamura K. (2014). Nat. Commun..

[cit27] Ventosa E., Schuhmann W. (2015). Phys. Chem. Chem. Phys..

[cit28] McCreery R. L., Cline K. K., McDermott C. A., McDermott M. T. (1994). Colloids Surf., A.

[cit29] Domi Y., Ochida M., Tsubouchi S., Nakagawa H., Yamanaka T., Doi T., Abe T., Ogumi Z. (2011). J. Phys. Chem. C.

[cit30] Tsubouchi S., Domi Y., Doi T., Ochida M., Nakagawa H., Yamanaka T., Abe T., Ogumi Z. (2012). J. Electrochem. Soc..

[cit31] Yamada Y., Miyazaki K., Abe T. (2010). Langmuir.

[cit32] Persson K., Sethuraman V. A., Hardwick L. J., Hinuma Y., Meng Y. S., Van Der Ven A., Srinivasan V., Kostecki R., Ceder G. (2010). J. Phys. Chem. Lett..

[cit33] Winter M., Novák P., Monnier A. (1998). J. Electrochem. Soc..

[cit34] Rice R. J., McCreery R. L. (1989). Anal. Chem..

[cit35] Collins J., Gourdin G., Foster M., Qu D. (2015). Carbon.

[cit36] Placke T., Siozios V., Schmitz R., Lux S. F., Bieker P., Colle C., Meyer H. W., Passerini S., Winter M. (2012). J. Power Sources.

[cit37] Ogihara N., Igarashi Y., Kamakura A., Naoi K., Kusachi Y., Utsugi K. (2006). Electrochim. Acta.

[cit38] Domi Y., Ochida M., Tsubouchi S., Nakagawa H., Yamanaka T., Doi T., Abe T., Ogumi Z. (2012). J. Electrochem. Soc..

[cit39] Domi Y., Doi T., Ochida M., Yamanaka T., Abe T., Ogumi Z. (2016). J. Electrochem. Soc..

[cit40] Nakagawa H., Domi Y., Doi T., Ochida M., Tsubouchi S., Yamanaka T., Abe T., Ogumi Z. (2012). J. Power Sources.

[cit41] Levi M. D., Aurbach D. (1997). J. Electroanal. Chem..

[cit42] Ogumi Z., Inaba M. (1998). Bull. Chem. Soc. Jpn..

[cit43] Flandrois S., Simon B. (1999). Carbon.

[cit44] Bülter H., Peters F., Schwenzel J., Wittstock G. (2014). Angew. Chem., Int. Ed..

[cit45] Rehnlund D., Ihrfors C., Maibach J., Nyholm L. (2018). Mater. Today.

[cit46] Heien M. L. A. V., Johnson M. A., Wightman R. M. (2004). Anal. Chem..

[cit47] Rodeberg N. T., Sandberg S. G., Johnson J. A., Phillips P. E. M., Wightman R. M. (2017). ACS Chem. Neurosci..

[cit48] Levi M. D., Levi E. A., Aurbach D. (1997). J. Electroanal. Chem..

[cit49] Li J., Xiao X., Yang F., Verbrugge M. W., Cheng Y.-T. (2011). J. Phys. Chem. C.

[cit50] Inaba M., Siroma Z., Kawatate Y., Funabiki A., Ogumi Z. (1997). J. Power Sources.

[cit51] Katagiri G., Ishida H., Ishitani A. (1988). Carbon.

[cit52] NuLi Y., Yang J., Jiang Z. (2006). J. Phys. Chem. Solids.

[cit53] Hernández-Burgos K., Rodríguez-Calero G. G., Zhou W., Burkhardt S. E., Abruña H. D. (2013). J. Am. Chem. Soc..

[cit54] Burgess M., Hernández-Burgos K., Cheng K. J., Moore J. S., Rodríguez-López J. (2016). Analyst.

[cit55] Fryling M. A., Zhao J., McCreery R. L. (1995). Anal. Chem..

[cit56] Ray K., McCreery R. L. (1997). Anal. Chem..

[cit57] Aurbach D., Levi M. D., Levi E., Schechter A. (1997). J. Phys. Chem. B.

[cit58] Domi Y., Doi T., Nakagawa H., Yamanaka T., Abe T., Ogumi Z. (2016). J. Electrochem. Soc..

[cit59] Smith R. B., Khoo E., Bazant M. Z. (2017). J. Phys. Chem. C.

[cit60] Ritzert N. L., Rodríguez-López J., Tan C., Abruña H. c. D. (2013). Langmuir.

[cit61] White S. H., Twardoch U. M. (1987). J. Appl. Electrochem..

[cit62] Kiani A., Alpuche-Aviles M. A., Eggers P. K., Jones M., Gooding J. J., Paddon-Row M. N., Bard A. J. (2008). Langmuir.

[cit63] Liu Q., Du C., Shen B., Zuo P., Cheng X., Ma Y., Yin G., Gao Y. (2016). RSC Adv..

[cit64] Yamada Y., Yaegashi M., Abe T., Yamada A. (2013). Chem. Commun..

[cit65] Sivakkumar S. R., Nerkar J. Y., Pandolfo A. G. (2010). Electrochim. Acta.

[cit66] Attia P. M., Das S., Harris S. J., Bazant M. Z., Chueh W. C. (2019). J. Electrochem. Soc..

[cit67] Zoski C. G. (2017). Curr. Opin. Electrochem..

[cit68] Sun H., Mei L., Liang J., Zhao Z., Lee C., Fei H., Ding M., Lau J., Li M., Wang C. (2017). Science.

[cit69] Ji B., Zhang F., Song X., Tang Y. (2017). Adv. Mater..

[cit70] Hwang J.-Y., Myung S.-T., Sun Y.-K. (2017). Chem. Soc. Rev..

[cit71] Kim H.-S., Cook J. B., Lin H., Ko J. S., Tolbert S. H., Ozolins V., Dunn B. (2017). Nat. Mater..

[cit72] Choi J. W., Aurbach D. (2016). Nat. Rev. Mater..

