# Supplementary material for: Probing the reversibility and kinetics of Li+ during SEI formation and (de)intercalation on edge plane graphite using ion-sensitive scanning electrochemical microscopy
Source: Chem Sci. 2019 Oct 8;10(46):10749–54. doi: 10.1039/c9sc03569a (PMC6993605; doi:10.1039/c9sc03569a)
Supplement: Supplementary file 1 [file SC-010-C9SC03569A-s001.pdf]

Electronic Supplementary Material (ESI) for Chemical Science.
This journal is © The Royal Society of Chemistry 2019

Probing the Reversibility and Kinetics of Li^+ during SEI formation and (De)Intercalation on Edge Plane Graphite using Ion-Sensitive Scanning Electrochemical Microscopy

Zachary T. Gossage,[‡] *Jingshu Hui,*[‡] *Yunxiong Zeng,*[‡] *Heriberto Flores-Zuleta,*[‡] *Joaquín Rodríguez-López*[‡,*]

‡ Department of Chemistry, University of Illinois at Urbana−Champaign, 600 S Mathews Ave., Urbana, Illinois 61801, United States

Corresponding Author
*To whom all correspondence should be addressed:
Prof. Rodríguez-López: joaquinr@illinois.edu (Email) and 217-300-7354 (Phone).

Table of Contents page

Section 1: Experimental Methods S3

Section 2: Description of COMSOL Simulations S5

Supplemental Table S1. Parameters for COMSOL simulations S6

Section 3: Supplemental Figures S7

Figure S1. HOPG edge plane fabrication procedure S7

Figure S2. Raman spectroscopy of unused HOPG edge plane S7

Figure S3. Approach curves to unused HOPG edge plane and LDPE and fittings S8

Figure S4. Approach curve to LDPE for SECM imaging of HOPG edge plane S8

Figure S5. SEM characterization of used regions of HOPG edge substrates S9

Figure S6. SECM image of HOPG for SEI and intercalation experiments with no mediator S9

Figure S7. HgDW microscopy and positioning for experiments with no mediator S10

Figure S8. PITT response during the first SEI formation cycle in 10 mM $LiPF_6$ S10

Figure S9. Raman spectrum of HOPG edge after DNPH treatment S11

Figure S10. SECM imaging and SEI formation on HOPG edge in 2M $LiBF_4$ S11

Figure S11. Change in HOPG response during SEI formation S12

Figure S12. Li intercalation of HOPG edge in 2M $LiBF_4$ S12

Figure S13. Comparison of i_{sp} during intercalation and deintercalation S13

Figure S14. Measured HOPG charge during cycling in the Intercalation region S13

Figure S15. Comparison of HgDW measurements at different times S14

Figure S16. Analysis of the amalgamation current S14

References S15

Section 1: Experimental Methods

Chemicals and Materials

Lithium hexafluorophosphate ($LiPF_6$), lithium perchlorate ($LiClO_4$) and lithium tetrafluorborate ($LiBF_4$) were all ≥99.99% purity and purchased from Sigma Aldrich as the lithium source and/or electrolyte. For additional supporting electrolyte, we used tetrabutyl ammonium hexafluorophosphate ($TBAPF_6$ (TCI, >98%)). *N,N,N′,N′*-tetramethyl-*p*-phenylenediamine (TMPD, 99%, Sigma Aldrich) and ferrocene (Fc, 98%, Sigma Aldrich) were used as received as redox mediators for SECM imaging and positioning. All electrolyte solutions were prepared with 1:1 (by volume) mixtures of propylene carbonate (PC, anhydrous, 99.7%, Sigma Aldrich) and ethylene carbonate (EC, anhydrous, 99%, Sigma Aldrich). 2,4-dinitrophenyhydrazine (DNPH, 97%, Sigma Aldrich) was used as a Raman probe as received. Platinum ultramicroelectrodes (UME (Goodfellow, purity 99.9%, 12.5 µm radius)) for initial SECM imaging were prepared as described in previous reports.[1] All purchased chemicals were used as received without further purification.

HOPG substrate preparation

Highly oriented pyrolytic graphite (HOPG, brand grade SPI-2 from SPI supplied) and solid slabs of flexible low-density polyethylene (LDPE, 12" x 12" x 1/4" sheet from McMaster-Carr) were used for substrate preparation. The HOPG was sealed between two pieces of LDPE with a vacuum oven at 110 °C for 2 hours and cooled under ambient conditions. The HOPG edge plane was then exposed by cutting and polished to flat surface with 1-5 µm SiC sandpaper. The substrate was rinsed thoroughly with PC before SECM experiments. All Raman measurements were conducted with a 532 nm laser using a Nanophoton Laser Raman Microscope RAMAN-11.

HgDW preparation

The HgDW probes were prepared as described previously.[2] In brief, Pt UMEs were prepared using standard protocols.[1] They were sharpened and polished using sandpaper (P4000) and alumina paste (1 µm), respectively. The probes were etched electrochemically in an aqueous solution of 30 v.% calcium chloride (99%, Sigma Aldrich), and 10 v.% hydrochloric acid (Macron) with an AC waveform of 2.7 V using a variable autotransformer and graphite rod as the counter electrode. Sonication was used during the etching procedure and afterward in clean HPLC-grade water to clean the probes and remove residual etching solution. Next, Hg was electrodeposited from 5 mM mercury (II) nitrate monohydrate (≥99.99%, trace metals basis, Sigma Aldrich), and 100 mM potassium nitrate (>99%, Fisher Scientific) to refill the well. Upon filling the well, the probe was examined under an optical microscope and a glass coverslip was used to press the droplet into a flat disc. Probes were then transferred into the glovebox for SECM experiments by gradual, low pressure vacuum cycles in the antechamber to remove water and oxygen.

SECM experiments

All electrochemical measurements were performed using a CHI920D Scanning Electrochemical Microscope (CH Instruments, Inc.) inside an oxygen and moisture-free glovebox. The HOPG substrates were assembled in a standard SECM cell, transferred into the glovebox and rinsed three times with fresh PC. For the first substrate, we replaced the PC with 15 mM Fc and 0.1 M $LiClO_4$ in PC:EC. We leveled the HOPG with a Pt UME and collected initial SECM images.

Thereafter, we ran multiple LSV scan from 3.3 to 0.5 V *vs*. Li^+/Li. We used a Pt wire as the counter electrode and a polished Ag wire as a quasi-reference. All potentials were converted to the Li^+/Li scale using standard potential of the redox mediator (Fc or TMPD) and of Li^+ amalgamation-stripping. After several scans, we reapproached the HOPG with the Pt UME and reimaged the same region. For the second substrate used in the intercalation experiments, we replaced the PC with 10 mM TMPD, 10 mM $LiPF_6$, 100 mM $TBAPF_6$ in PC:EC. We leveled and imaged the substrate using the same protocol as the first sample. Thereafter we replaced the Pt UME with a HgDW (12.5 µm radius), approached again to the surface and positioned the probe above the center of the HOPG substrate. We approached to the surface, retracted and rinsed the cell three times with fresh PC. We refilled the cell with 10 mM $LiPF_6$ and 100 mM $TBAPF_6$ and repositioned the probe close to the HOPG substrate. We continually cycled the probe with cyclic voltammetry to quantify Li^+ in the vicinity of the probe. While collecting information at the probe, we applied potential steps to the substrate (~16 s each) in 100 mV increments between 3.0 and 0.6 V vs Li. After six cycles we stepped the substrate further negative and decreased the step size to 50 mV.

DNPH modification of the HOPG surface

Following previous protocols,[3, 4] we prepared a 10 mM DNPH solution in ethanol (with 1% HCl). We degassed the solution and brought it to a boil while stirring. Next, we submerged a fresh HOPG substrate, turned off heat and continued degassing and stirring for 2 hours while the reaction proceeded. We removed the substrate, rinsed thoroughly with ethanol and submerged it into a solution of 0.1M KOH in ethanol for 10 minutes. Finally we rinsed again with ethanol, allowed the sample to dry and conducted Raman spectroscopy under ambient conditions.

COMSOL simulations

Simulations were completed using the Transport of Diluted Species module within COMSOL Multiphysics 4.4. For our simulations, we utilized a closed-boundary, 2-D axisymmetric geometry resembling the experimental setup and Fick's laws to govern diffusion. We applied Butler-Volmer to evaluate Li^+ intercalation kinetics of the substrate domain. Further details are provided in the Supplemental Information Section 2 below.

Section 2: Description of COMSOL Simulations

Simulations were completed using the Transport of Diluted Species module within COMSOL Multiphysics 4.4, using Fick's laws for diffusion. For simulation of the intercalation process (Figure 3, main text), we used a 2D axisymmetric geometry representing a radial cross section of the HgDW probe positioned near the HOPG electrode (Figure 3, main text and below). Three active domains were defined: 1) Amalgam, 2) HOPG, and 3) Solution. All parameters used in the simulations are listed in Supplemental Table 1 with reference values. The Amalgam domain and its Flux boundary with the Solution domain involved consumption of species (M^+) at the Flux boundary to produce reduced species (M(Hg)) that could diffuse freely into the Amalgam domain. Likewise, the HOPG domain was defined the same way as the Amalgam domain but with its own parameters and Flux boundary defined by Butler-Volmer. The potential at the Amalgam domain Flux boundary was controlled based on a sweeping potential to simulate cyclic voltammetry at the probe. For each simulation the potential applied to the HOPG domain Flux boundary, subE, was maintained at a constant value. Open boundaries were set to bulk conditions. Most values collected from the literature agreed with our simulations. We note the largest discrepancies involve those surrounding the HgDW (e.g. k^0, Dred, $\alpha_{Hg\text{-}Li}$). HgDW probes are sensitive to the electrolyte environment, and contaminants, especially at the Hg surface, can affect the overall probe response. However, even non-ideal probes can be quite stable throughout measurements. We used the parameters that fit best for multiple curves and considered the substrate response for interpretation.

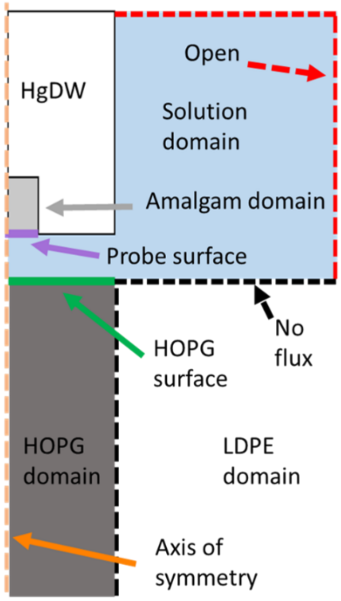

Hg domain B-V (1)

$$k_f = k^0 * \exp(-\alpha_{Hg\text{-}Li} * n * f * (Eapp - E^0))$$

$$k_b = k^0 * \exp((1 - \alpha_{Hg\text{-}Li}) * n * f * (Eapp - E^0))$$

HOPG domain B-V (2)

$$k_{fLi} = k^0_{Li} * \exp(-\alpha_{HOPG\text{-}Li} * n * f * (subE - sE^0))$$

$$k_{bLi} = k^0_{Li} * \exp((1 - \alpha_{HOPG\text{-}Li}) * n * f * (subE - sE^0))$$

Supplemental Table S1. Parameters for COMSOL simulations.

Parameter	Variable	Simulated value	Reported values
HgDW electrode radius	R	12.5×10^{-6} [m]	-
HgDW depth	H	37.5×10^{-6} [m]	-
Length of HgDW	h2	62.5×10^{-6} [m]	-
HgDW:glass ratio	RG	33.75×10^{-6} [m]	-
HOPG electrode radius	Hedge	30×10^{-6} [m]	-
HOPG depth	Dedge	100×10^{-6} [m]	-
HOPG-HgDW distance	D	2.5×10^{-6} [m]	-
Cell width	Wcell	500×10^{-6} [m]	-
Potential sweep rate – HgDW	Nu	1 [V/s]	-
Potential sampling interval	Eint	0.001 [V]	-
Time sampling interval	Tint	Eint/nu	-
Positive potential limit	Eox	2.0 [V]	-
Negative potential limit	Ered	0.4 [V]	-
Potential applied at tip	Eapp	pw1(t)	-
Time, floating parameter	T	0 (s)	-
Bulk [M^+]	ox^0	10 [mol/m^3]	-
Electron transfer coefficient - M^+ -> M(Hg)	$\alpha_{Hg\text{-}Li}$	0.25	0.7[5]
Reduction potential - M^+ -> M(Hg)	E^0	0.96 [V]	-
Apparent rate constant - M^+ -> M(Hg)	k^0	0.0005 [cm/s]	0.01[5], 0.008[6]
Forward rate constant – HgDW	k_f	B-V(1) *See above	-
Backward rate constant – HgDW	k_b	B-V(1) *See above	-
Diffusion coefficient – M^+ in solution	Dox	1.7×10^{-6} [cm^2/s]	1.7[6], 2.4[5] for PC
Diffusion coefficient – M in Hg	Dred	5×10^{-6} [cm^2/s]	9.2[7]
Electron transfer coefficient – M^+ intercalation	$\alpha_{HOPG\text{-}Li}$	0.08	0.1[8]
Standard reduction potential - M^+ intercalation	sE^0	0.09 [V]	0.22,0.13, 0.086[9]
Apparent rate constant – M^+ intercalation	k^0_{Li}	1×10^{-4} [cm/s]	(10^{-4})[8], (10^{-7})[9]
Forward rate constant – M^+ intercalation	k_{fLi}	B-V(2) *See above	-
Backward rate constant – M^+ deintercalation	k_{bLi}	B-V(2) *See above	-
Diffusion coefficient – M in HOPG	Dedge	3×10^{-10} [cm^2/s]	2 to 3.4[9]
Applied substrate potential	subE	3 to -0.5 [V]	-
Moles of electrons per mole M^+ reduction	*N*	1	-
Faraday's constant	F	96485.3 [C/mol]	-
Universal gas constant	R	8.314 [J/(mol * K)]	-
Temperature	T	298.15 [K]	-
F/(R*T)	*F*	38.9 [1/V]	-

Section 3: Supplemental Figures

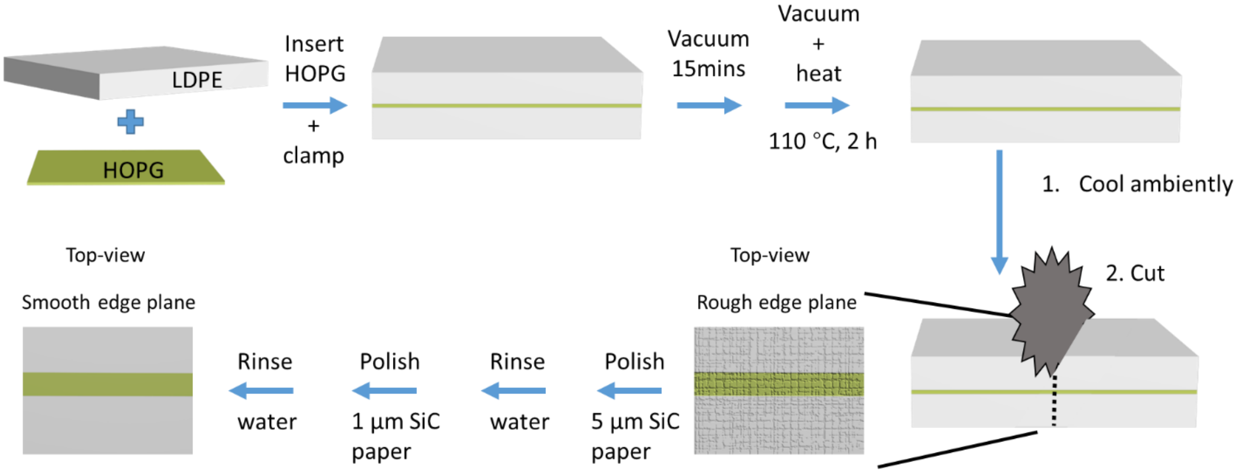

Figure S1. HOPG edge plane fabrication procedure.

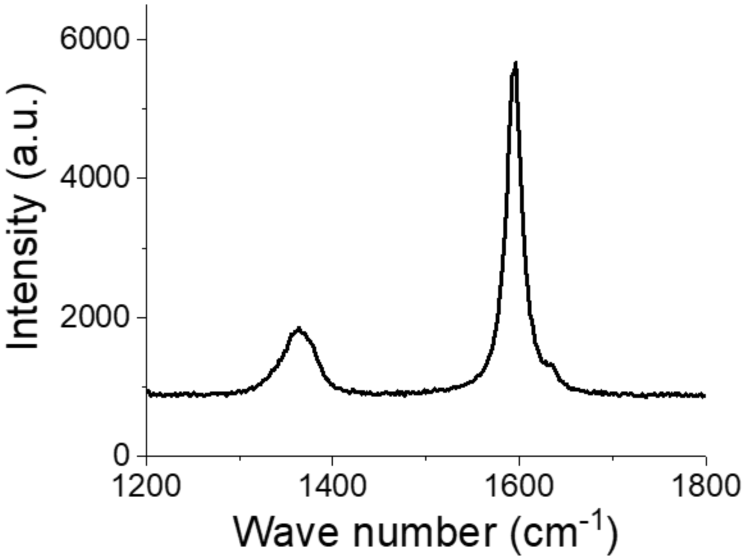

Figure S2. Raman spectroscopy of unused HOPG edge plane electrode.

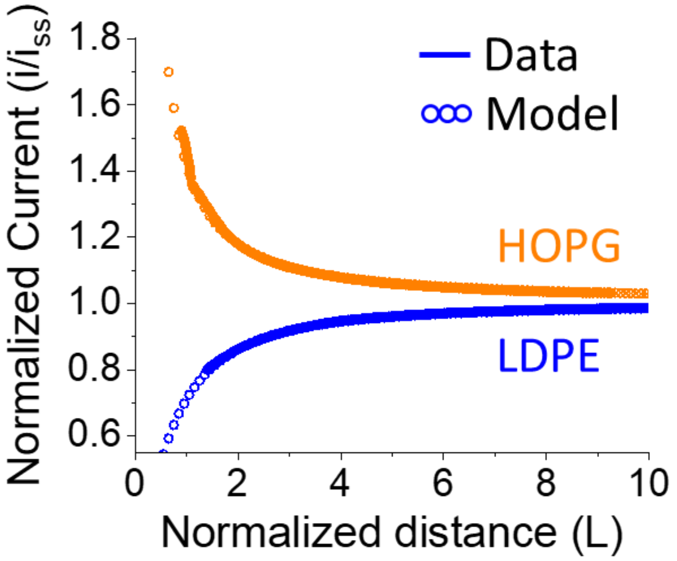

Figure S3. Approach curves to HOPG edge and surrounding LDPE. Fittings based on Cornut and Lefrou.[10, 11]

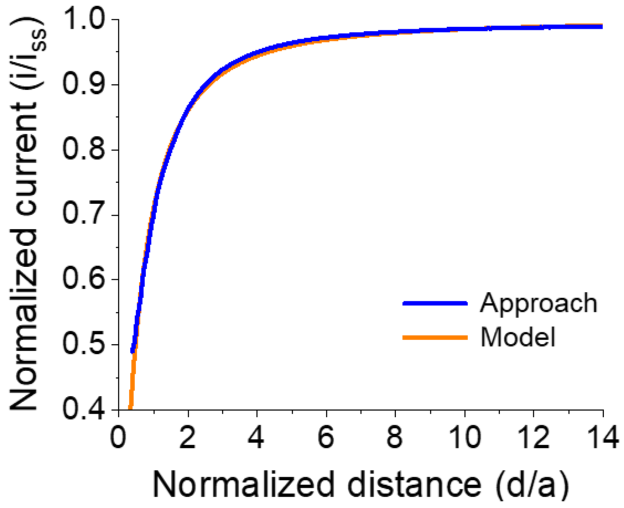

Figure S4. Approach to LDPE. The probe was stopped at 4.75 µm from the LDPE for SECM imaging of an unused HOPG edge.

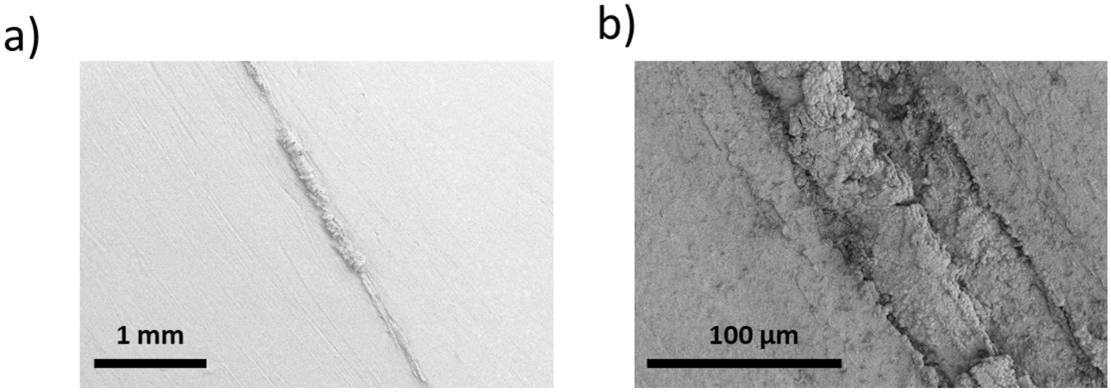

Figure S5. SEM characterization of fresh and used regions on HOPG edge samples after intercalation experiments. a) and b) show used regions with large protrusions, cracking and holes

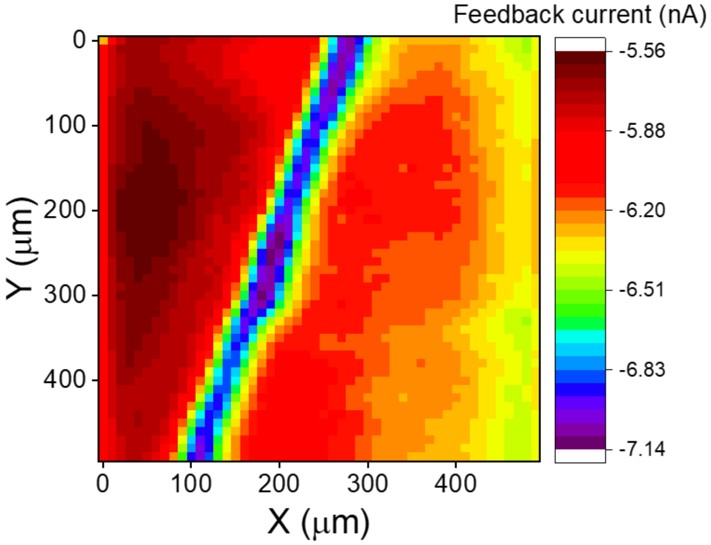

Figure S6. SECM image of HOPG edge for first SEI and intercalation data set. The image was collected in 10 mM TMPD, 10 mM $LiPF_6$, 100 mM $TBA.PF_6$ in PC:EC (1:1).

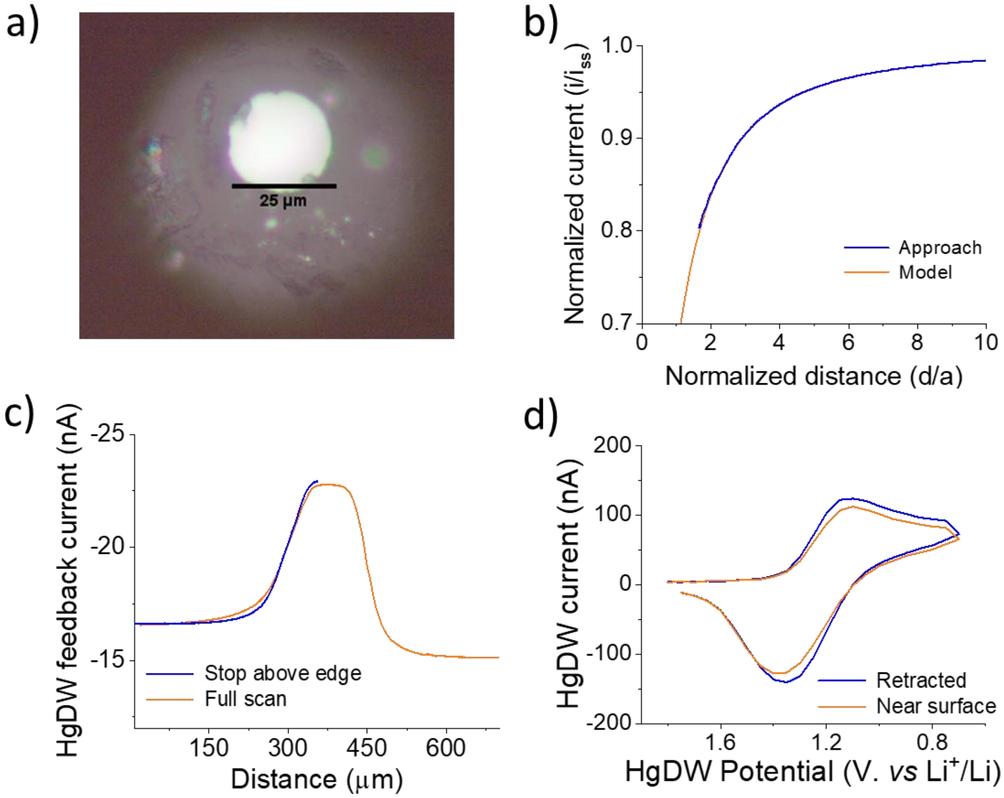

Figure S7. HgDW probe and positioning for SEI and intercalation measurements. a) Optical microscopy of HgDW after pressing. b) Approach curve to the LDPE portion of the substrate. c) Positioning the HgDW above the HOPG edge using linescans in the X direction. d) Probe response near the surface and retracted ~ 60 µm.

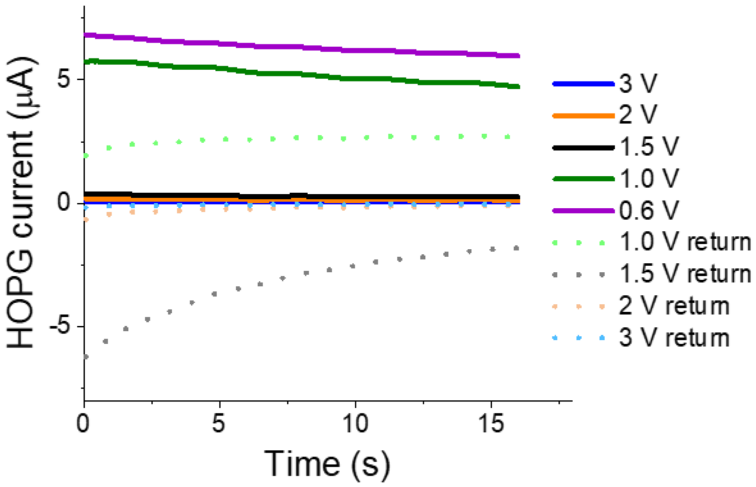

Figure S8. HOPG transients during the first SEI formation cycle at select potentials. The electrolyte was 10 mM $LiPF_6$, 100 mM $TBA.PF_6$ in PC:EC.

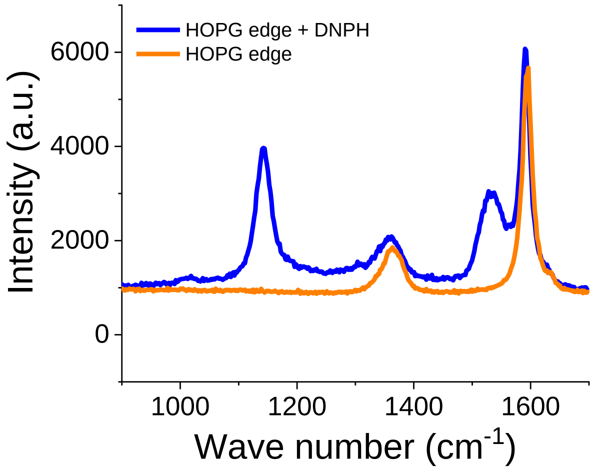

Figure S9. Raman spectrum of HOPG edge plane samples with and without reaction with DNPH.

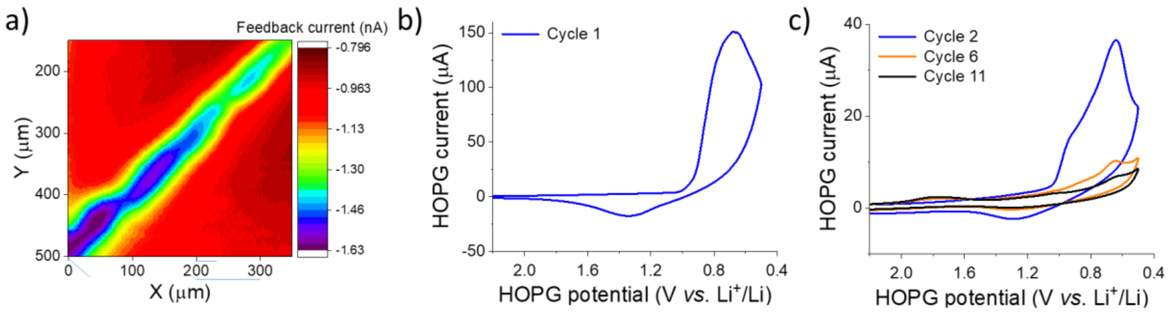

Figure S10. SEI formation on HOPG edge in 2M $LiBF_4$. a) SECM imaging before SEI formation. b) First SEI formation cycle. c) Cycling the HOPG edge sample to form the SEI at 10 mV/s.

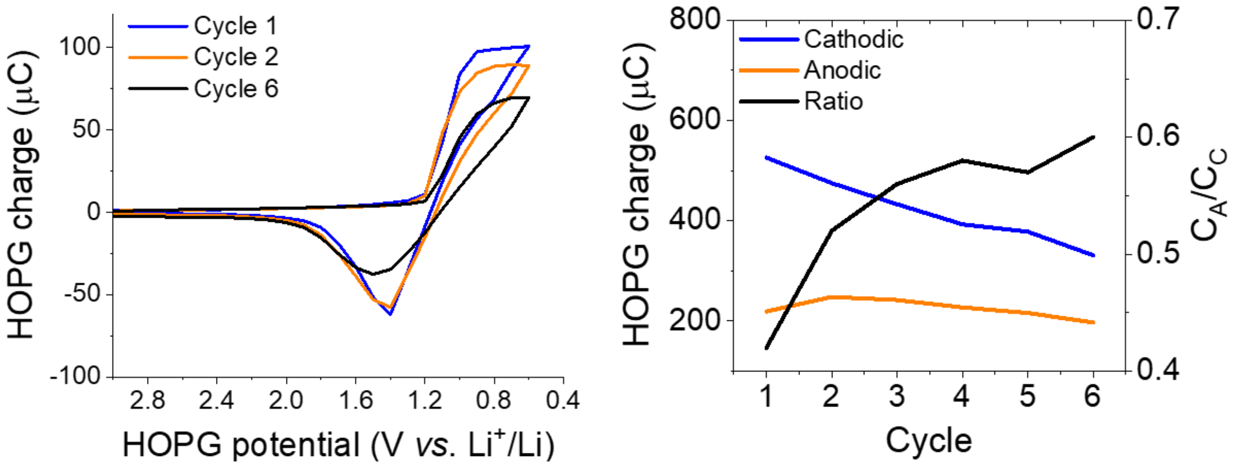

Figure S11. Change in HOPG response during SEI formation. a) Comparison of HOPG charge at each potential for the first six cycles in the SEI region. b) Comparison of the total added charge for the cathodic and anodic processes during cycling.

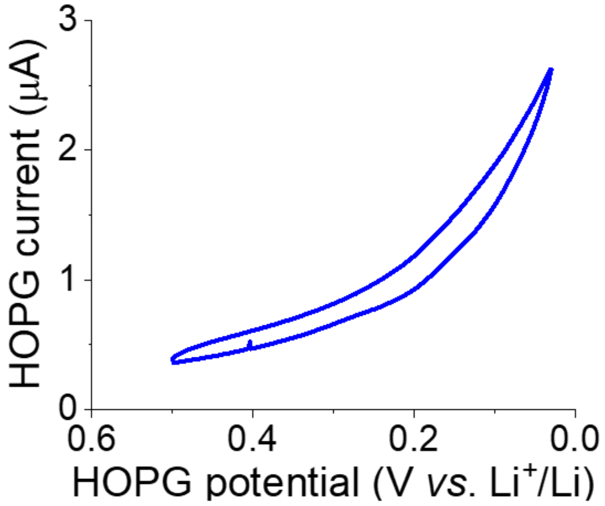

Figure S12. Intercalation region on the HOPG edge in 2M $LiBF_4$. The substrate was scanned at 200 μV/s.

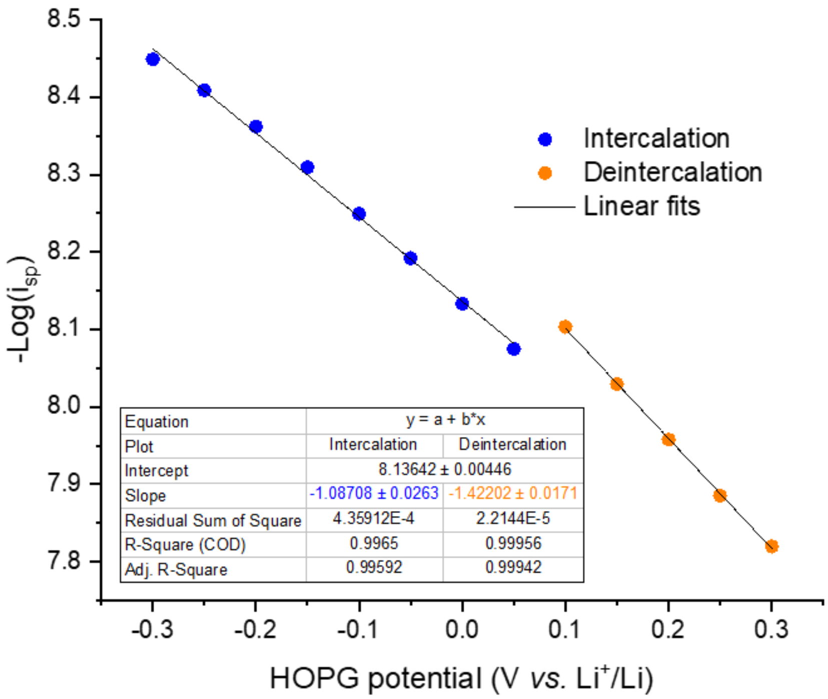

Figure S13. Comparison of i_{sp} during intercalation and deintercalation during cycle 8.

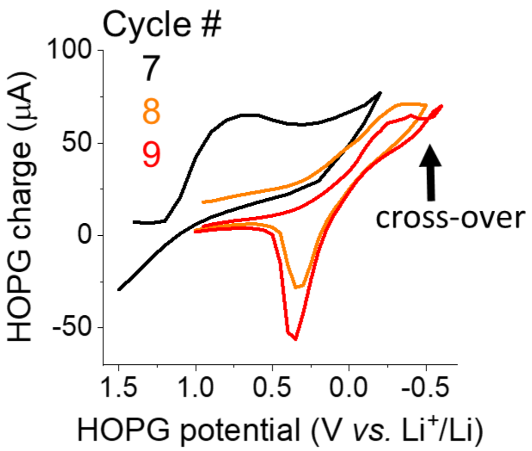

Figure S14. Measured HOPG charge during cycling in the Intercalation region. Cross-over indicating plating and nucleation occurred in cycle 9.

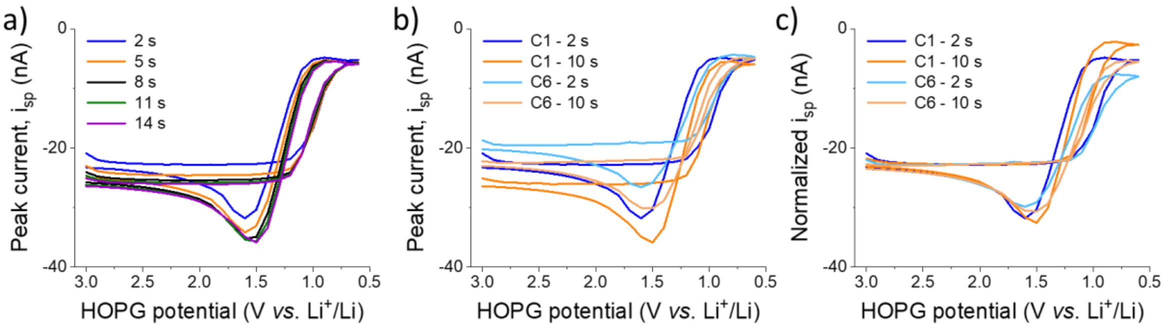

Figure S15. Stripping at later times for all potentials and normalization. a) Processed data for each HgDW cycle with peaks collected at different times during the first SEI formation cycle (C1). b) Comparison of SEI formation cycles 1 (C1) and 6 (C6) for HgDW data at different times. c) Comparison of normalized data.

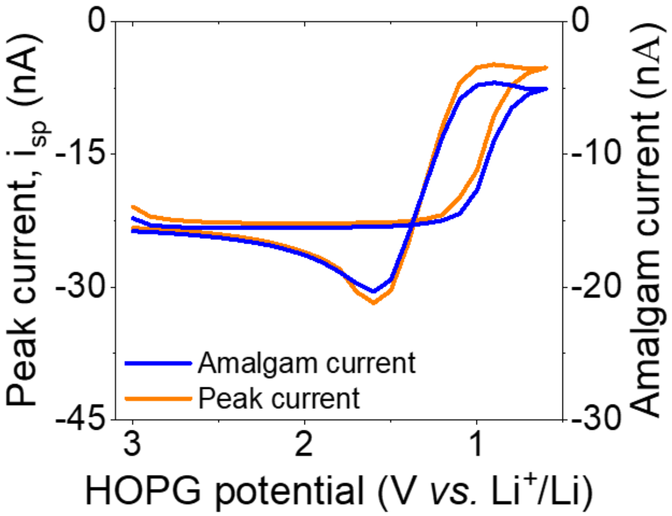

Figure S16. Amalgamation data processed for the first SEI formation cycle.

References

1. Bard, A. J.; Fan, F. R. F.; Kwak, J.; Lev, O., Scanning electrochemical microscopy. Introduction and principles. *Analytical Chemistry* **1989,** *61* (2), 132-138.
2. Barton, Z. J.; Rodríguez-López, J. n., Fabrication and demonstration of mercury disc-well probes for stripping-based cyclic voltammetry scanning electrochemical microscopy. *Analytical chemistry* **2017,** *89* (5), 2716-2723.
3. Fryling, M. A.; Zhao, J.; McCreery, R. L., Resonance Raman observation of surface carbonyl groups on carbon electrodes following dinitrophenylhydrazine derivatization. *Analytical Chemistry* **1995,** *67* (5), 967-975.
4. Ray, K.; McCreery, R. L., Spatially resolved Raman spectroscopy of carbon electrode surfaces: observations of structural and chemical heterogeneity. *Analytical Chemistry* **1997,** *69* (22), 4680-4687.
5. Hills, G. J.; Peter, L. M., Electrode kinetics in aprotic media. *Journal of Electroanalytical Chemistry and Interfacial Electrochemistry* **1974,** *50* (2), 175-185.
6. Cronnolly, C.; Pillai, K. C.; Waghorne, W. E., Electrode kinetic studies of the Zn2+/Zn (Hg) and Li+/Li (Hg) couples in dimethylsulphoxide+ propylene carbonate solvent systems. *Journal of electroanalytical chemistry and interfacial electrochemistry* **1986,** *207* (1-2), 177-187.
7. Kozin, L. F.; Hansen, S. C., *Mercury handbook: chemistry, applications and environmental impact*. Royal Society of Chemistry: 2013.
8. Ritzert, N. L.; Rodríguez-López, J. n.; Tan, C.; Abruña, H. c. D., Kinetics of interfacial electron transfer at single-layer graphene electrodes in aqueous and nonaqueous solutions. *Langmuir* **2013,** *29* (5), 1683-1694.
9. Levi, M. D.; Aurbach, D., The mechanism of lithium intercalation in graphite film electrodes in aprotic media. Part 1. High resolution slow scan rate cyclic voltammetric studies and modeling. *Journal of Electroanalytical Chemistry* **1997,** *421* (1-2), 79-88.
10. Cornut, R.; Lefrou, C., A unified new analytical approximation for negative feedback currents with a microdisk SECM tip. *Journal of Electroanalytical Chemistry* **2007,** *608* (1), 59-66.
11. Lefrou, C., A unified new analytical approximation for positive feedback currents with a microdisk SECM tip. *Journal of Electroanalytical Chemistry* **2006,** *592* (1), 103-112.
